# Editorial: Reviews and advances in inflammatory diseases and the tumor necrosis factor

**DOI:** 10.3389/fcell.2024.1399804

**Published:** 2024-04-09

**Authors:** Cristian R. Smulski

**Affiliations:** Medical Physics Department, Bariloche Atomic Centre (CNEA, CONICET), San Carlos de Bariloche, Argentina

**Keywords:** TNF, inflammation, signalling, cell death, disease

## Summary

Tumor necrosis factor alpha (TNF) is a key modulator of immune responses. Its ability to trigger pro-survival and apoptotic or necroptotic signaling pathways has long been the subject of intense research. However, its role in inflammatory diseases is far from being fully understood. The outcome of signaling, following ligand binding, results from a tightly and timely interplay of phosphorylation and ubiquitination events that builds up different intracellular signaling complexes. Disruption or modification of these signaling complexes in a given environment could result in severe inflammatory responses. The “Reviews and Advances in Inflammatory diseases and the Tumor Necrosis Factor” Research Topic provides new insights into complex regulatory mechanisms leading to inflammation.

## A brief background

### TNF superfamily

Ligands and receptors of the tumor necrosis factor superfamily (TNF/TNFR -SF) comprise 19 ligands and 30 receptors. TNF family ligands share a common structural motif that mediates trimerization of the extracellular domain, called the TNF homology domain (Bodmer et al., 2002). TNF family receptors share a common structural motif on the extracellular region that mediates ligand binding and, in some cases, receptor oligomerization named cysteine rich domain (Chan et al., 2000; Smulski et al., 2013; 2017; Kucka and Wajant, 2020). In addition, the transmembrane domain has been shown to mediate receptor oligomerization for some TNFRs (Fu et al., 2016; Nadezhdin et al., 2016; Pan et al., 2019; Sica and Smulski, 2021; Smulski et al., 2022). The intracellular region distinguish two kinds of receptors: those that recruits TNF receptor associated factors (TRAFs) and those containing a Death Domain (DD) able to trigger cell death as TNFR1.

### TNF signaling

TNF-TNFR1 constitutes one of the most studied ligand-receptor pair of the family. Signaling outcome ranges from NF-kB and MAPK activation to apoptosis or necroptosis (Ting and Bertrand, 2016). Binding of TNF to TNFR1 leads to the assembly of the TNFR1-associated signaling complex I. This complex contain the adaptor proteins RIP1 (RIPK1) and TRADD (Micheau and Tschopp, 2003). Then, TRADD recruits the adaptor proteins TRAF2 and TRAF5, which enables the engagement of the E3 ubiquitin ligases c-IAP1 and 2 with the subsequent ubiquitination of different components of complex I (Bertrand et al., 2008; Mahoney et al., 2008; Varfolomeev et al., 2008; Dynek et al., 2010). Polyubiquitin chains provides a docking platform for the assembly of the IKK complex leading to activation of NF-κB and MAPK signaling pathways. These signals leads to the expression of pro-inflammatory cytokines, such as interleukin 6 and 8, and pro-survival proteins like c-IAP2 and the caspase-8 inhibitor cellular FLICE inhibitory protein (cFLIP) (Scheidereit, 2006). Alternatively a cytosolic complex II leading to cell death originates from the dissociation of TRADD and RIP1 from complex I and their association with FADD and caspase 8. Notably, different cell death checkpoints actively repress TNF cytotoxicity and their inactivation will lead to different cell death programs such as RIP1 dependent apoptosis, RIP1 dependent pyroptosis (GSDMD-dependent), RIP1 independent apoptosis or RIP1 dependent necroptosis (MLKL-dependent) (van Loo and Bertrand, 2023).

### Inflammation

The importance of TNF as a key inflammation driver has been confirmed by the identification of patients with severe inflammatory diseases carrying defects in TNF signaling components (Manthiram et al., 2017; Oda et al., 2019). Indeed, there are a plethora of tightly regulated molecules involved in different signaling outcomes (van Loo and Bertrand, 2023) and thus, disruption of these events can lead to disease, for example:

- Activation of NF-kB and MAPK signaling pathways by TNF, lead to the transcriptional upregulation of genes encoding proinflammatory mediators, such as cytokines and chemokines.
- Triggering of lytic forms of cell death (necrosis, pyroptosis and necroptosis) release damage-associated molecular patterns that activate proinflammatory gene expression in bystander cells.
- Availability of intracellular signaling components regulated by other signals or pathogens can shift TNF signaling outcome.
- Induction of cell death on epithelial cells creates a breach for microorganisms with the subsequent increase in pathogen-associated molecular patterns that activate proinflammatory gene expression.

## Inflammatory diseases and the tumor necrosis factor

In mice, disruption of the gene that codes for Tristetraprolin (TTP) led to severe arthritis, autoimmunity, cachexia and dermatitis mainly due to excessive TNFα levels in the affected tissues. Stedile et al., has shown a novel regulatory mechanism mediated by TTP, which keeps low TNFα levels, leading to survival of mammary progenitor cells. In addition, TTP expression is required for lactation maintenance (Goddio et al., 2018). Authors showed that survival of the stem-like cells is compromised by increased levels of inflammatory cytokines. Therefore, expression of TTP in the mammary gland is necessary for the maintenance of the progenitor cell compartment by keeping low TNFα levels. Notably, in humans, TNF-α inhibitors may be a potential treatment for at least some types of Mastitis caused by infection or autoimmunity in women of fertile age (Goulabchand et al., 2020; Chiu et al., 2022).

Notably, TNF can bind to a second receptor of the family, TNFR2, whose immune function differs from TNFR1. While TNFR1 associates strongly with both membrane-bound and soluble TNF, TNFR2 shows higher affinity for membrane-bound TNF (Grell et al., 1995). Liganded TNFR2 recruit TRAF2 along with the partners cIAP1 and cIAP2 leading to activation of the classical NFκB pathway. Siegmund et al., has shown that engagement of TNFR2 and of another TNFR family member Fn14, can limit the availability of adaptor molecules recruited to TNFR1. Authors has shown that engagement of TNFR2 or Fn14 inhibits TNFR1-induced RIPK1-mediated effects due to depletion of TRAF2 and cIAP1/2. Future studies will determine to which extent TNFR2/Fn14-TNFR1 crosstalk could contribute to pathophysiological scenarios in which TNFR1-induced cell death plays a crucial role.

As it was mentioned before, caspase 8 is a key component for ligand-induced cell death, not only for TNFR1 but also for other death receptors of the TNF superfamily (Fas, DR4, DR5). However, Vesla et al., showed that active caspase-8 is present in apoptotic and in non-apoptotic osteoblasts leading to the hypothesis that caspase-8 has a broader impact on osteoblastic differentiation. Indeed, osteogenic differentiation of caspase-8 deficient cells was inhibited as these cells displayed a decreased level of mineralization, lower activity of alkaline phosphatase and several changes in osteogenic-related gene expression. Additionally, caspase-8 deficiency inhibited the proliferation of osteoblastic cells. All together, these data provide new insight into the effects of caspase-8 on non-apoptotic osteogenic pathways.

Bronchiolitis induced by infection with the respiratory syncytial virus (RSV) is a significant contributor to infant morbidity and mortality. Infection of human airway epithelial cells with RSV leads to necroptosis (MLKL-dependent), a pro-inflammatory cell death that mediates the release of the alarmin high mobility group box 1 (HMGB1) (Hosakote et al., 2016; Sebina and Phipps, 2020). Now Simpson et al., take a closer look to the kinetics of HMGB1 release and the impact on cell death. They show a biphasic release of HMGB1 post infection. The early phase of HMGB1 release is cell death-independent and promotes the late phase of HMGB1 release via the activation of RAGE and occurs with cell death. As the inhibition of MLKL or targeting of HMGB1/RAGE pathway attenuates the release of pro-inflammatory HMGB1 and decreases viral load, this suggests that the pharmacological targeting of these pathways may be of benefit for the treatment of severe RSV bronchiolitis.

Overall, the current Research Topic has covered important landmarks of the macromolecular complexes and signaling pathways engaged by TNF/TNFR family members. Although other features must be understood for proper selective therapeutic intervention, understanding the complex function and signaling interplay can be exploited to design effective treatments, not only for inflammatory diseases but also for other pathologies such as cancer.
